# Environmental impact assessment of rice mill waste valorisation to glucose through biorefinery platform

**DOI:** 10.1038/s41598-023-28487-2

**Published:** 2023-09-07

**Authors:** Nurul Ain Abu-Bakar, Ahmad Muhaimin Roslan, Mohd Ali Hassan, Mohammad Hariz Abdul Rahman, Khairul Nadiah Ibrahim, Muhammad Daaniyall Abd Rahman, Rozyanti Mohamad

**Affiliations:** 1 Department of Bioprocess Technology, Faculty of Biotechnology and Biomolecular Sciences, Universiti Putra Malaysia, 43400 UPM Serdang, Selangor Malaysia; 2 Agrobiodiversity and Environment Research Centre, Malaysia Agriculture Research and Development Institute, Persiaran MARDI-UPM, 43400 Serdang, Selangor Malaysia; 3 Universiti Kuala Lumpur, Malaysian Institute of Chemical and Bioengineering Technology, Lot 1988 Bandar Vendor, Taboh Naning, 78000 Alor Gajah, Melaka Malaysia; 4 School of Business and Economics, Universiti Putra Malaysia, 43400 UPM Serdang, Selangor Malaysia; 5 Biopolymers and Derivatives Laboratory, Institute of Tropical Forestry and Forest Product, Universiti Putra Malaysia, 43400 UPM Serdang, Selangor Malaysia

**Keywords:** Biotechnology, Environmental sciences

## Abstract

Environmental impact assessment of glucose production from paddy milling waste, known as empty and partially filled paddy grain (EPFG) in Malaysia, was performed using life cycle assessment (LCA). Three scenarios were conducted based on system expansion of the process. The LCA was conducted using ReCiPe methodology at midpoint and endpoint levels. The results indicate that enzymatic hydrolysis phase is the hotspot in the conversion system due to enzyme production. In addition, the agriculture phase also contributed to negative impacts, especially towards climate change. An improved environmental load was observed in scenario 2 when all EPFG fractionation was utilised to replace fossil-based electricity. Sensitivity analysis showed an increase in glucose yield leads to reduced environmental impact. Thus, the LCA study suggests that the conversion process of EPFG could further benefit and improve the paddy industry waste management with low impact contribution to the environment compared to other feedstock used for glucose production.

## Introduction

Paddy is one of the important crops under the food subsector in Malaysia since it is the staple food for most of the population. Consequently, there is a constraint on paddy production to meet the demand since there are postharvest losses along the supply chain. Postharvest losses in paddy production commonly occur due to excessive exposure to extreme climate and improper management^1^. In 2017, the paddy postharvest losses in Malaysia were estimated at 28.5%, or equalled to 480,000 metric tons, resulting in a revenue loss of USD 134 million based on the paddy price of USD 279 per metric ton^2^. According to International Rice Research Institute (IRRI), around 5–21% of losses were from factory drying, storage, milling, and processing due to grain deterioration^1^. This is because paddy seed processing in the milling factories aims to produce quality seed varieties by removing undesired particles and damaged grains as waste. As a result, these damaged grains [referred to as empty and partially filled paddy grain (EPFG)] are abundant and take up space in the factory during paddy harvesting seasons. This EPFG waste was managed only by dumping it into the landfill, causing the loss of a natural resource for potential value-added product development.

Empty and partially filled paddy grain has the potential to become a biomass feedstock for glucose production as an intermediate for industrial bioproducts. Lignocellulosic biomass consist of cellulose, hemicellulose, and lignin which could be exploited as an energy sources^3^. The EPFG are characterised as lignocellulosic biomass that is rich in carbohydrates. The main composition in EPFG which play an important role in conversion to sugars is comprised of cellulose, hemicellulose, and starch^4^. Specifically, only starch and cellulose are further broken down to glucose by the means of biological or chemical processes.

The conversion of biomass to value-added products through the “sugar platform” is a part of the biorefinery concepts for the replacement of fossil-based products. The glucose acts as a source for further processing through different routes for industrial bioproduct development such as biopolymers, biochemical, and biofuels. Exploitation of lignocellulosic biomass for value-added products has been emphasized in the Twelfth Malaysian Plan, 2021–2025. With a total investment of RM 10 billion, the Malaysian government aims to accelerate the development of the biomass industry to boost economic growth. Moreover, the utilisation of lignocellulosic biomass for bioproducts is also recognised as a carbon–neutral process compared to products made from petroleum, thus making the overall process more environmentally friendly^5^.

Environmental aspects and impacts are crucial criteria related to biorefinery concepts in the biomass industry. The conversion of EPFG (as feedstock) to glucose may potentially contribute to a reduction of greenhouse gas (GHG) emissions and other environmental impacts on the paddy industry. To verify if managing EPFG by conversion to glucose products is more efficient and environmentally sustainable, it is necessary to evaluate the environmental impacts and provide information to policymakers. One such method is by conducting a Life cycle assessment (LCA). Life Cycle Assessment is a method to evaluate the environmental impacts of bioproducts especially in biorefineries systems^5,6^. The LCA concept assesses a product throughout its life cycle, starting from raw material extraction, production, transportation, product use, and disposal^7^.

However, most of the LCA studies published concerning biorefinery concepts focused more on the end-product of lignocellulosic biomass processes such as biofuels and biochemical^7,8^. The environmental impacts focusing on upstream processing for glucose production from biomass are scarce, especially in a developing country such as Malaysia^6,9^. Furthermore, the inventory and environmental assessment of LCA studies related to -generation biomass conversion to glucose or other sugar platforms were mostly published using European countries' data^6^. Therefore, the primary data in the LCA study uses the local data as much as possible to reflect the glucose production from EPFG in Malaysia's perspective. This may provide detailed information and results on waste management, which may impact the paddy industry and give better insight to policymakers. Currently, there are no detailed studies on the environmental impact of EPFG valorisation on glucose as value-added product in Malaysia. The environmental impact of LCA reports based on Malaysia inventory data by Abdul Rahman et al*.*^10^ and Harun et al*.*^11^ were mainly on paddy cultivation, while Shafie et al*.*^12^ reported on utilising paddy waste to generate electricity. Thus, this paper aims to evaluate the environmental impacts of EPFG for conversion to glucose as a more sustainable solution for waste management from paddy seed milling factories.

## Materials and methods

### Goal and scope of the study

This study adapted the ISO 14040/44 framework^13^ as the guidelines for the methodology. The goal of this study is to evaluate the environmental performance of the conversion process of EPFG as waste in Malaysia paddy industry into a value-added product; i.e. glucose, as an alternative for petroleum-based feedstock for paddy waste management. The scope of the study involved three different scenarios of glucose production as a platform for fermentation products. The study focused on identifying the hotspots that can be improved from the cradle-to-gate phase concurrent with evaluating the impacts of glucose production. The functional unit, defined as the quantified performance of a product for its use as a reference unit, was 1 tonne of glucose produced at the milling gate.

### Description of scenarios

**Figure 1:**
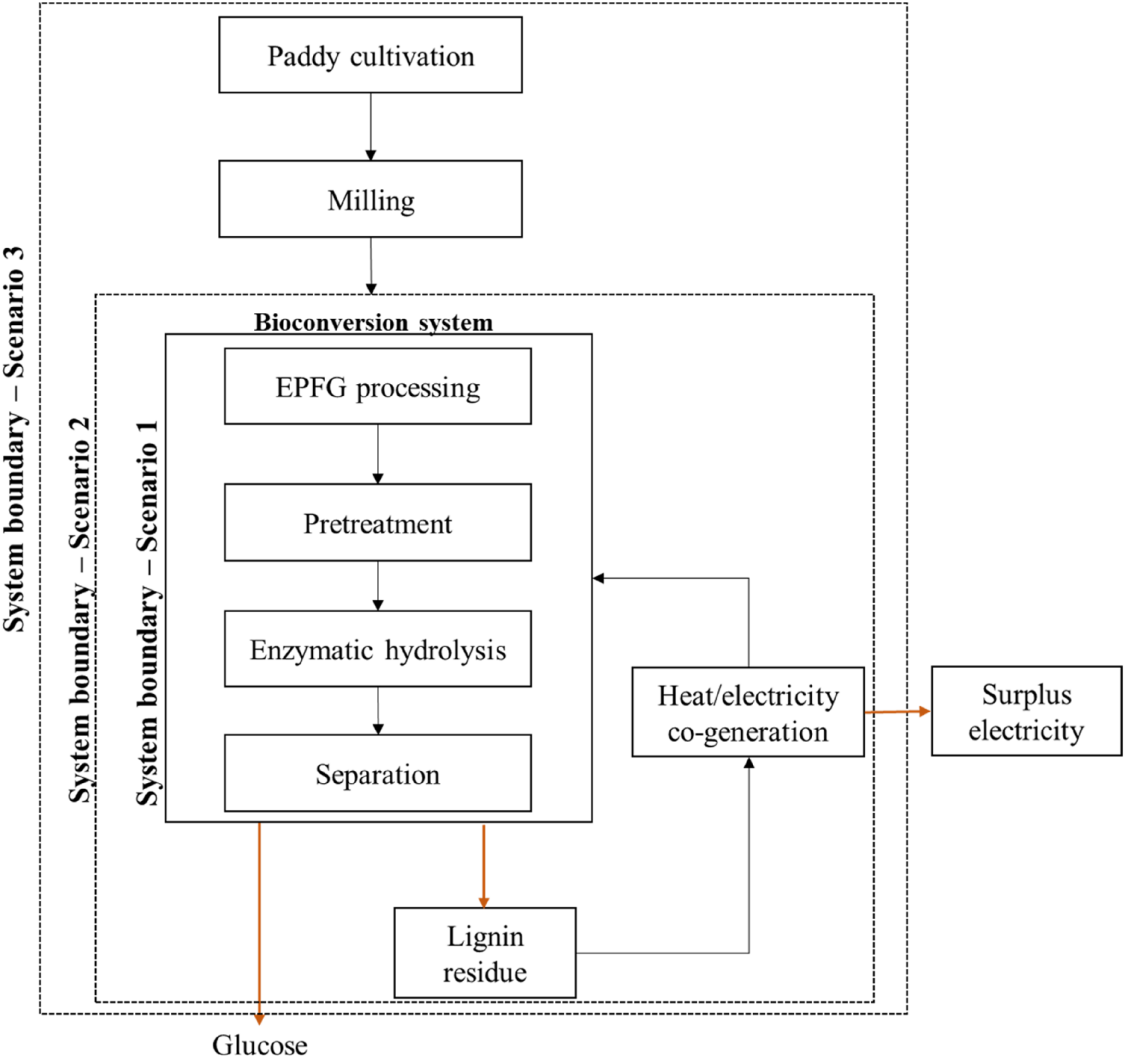
System boundary of this study.

Three different scenarios were performed in the LCA studies; (1) base case scenario (Scenario 1) involved the bioconversion of EPFG into glucose, (2) Scenario 2 considered the waste utilisation of the conversions process as combustion of lignin residue, and (3) Scenario 3 considered the production of EPFG from paddy cultivation, milling, and the conversion process to glucose. An overview of the LCA system boundary is presented in Fig. 1 for the three scenarios.

#### Scenario 1 (S1)—bioconversion of EPFG to glucose as value-added product

In this study, it is proposed that glucose production occurs on a single site within the paddy milling factory. The process-based conversions of EPFG to glucose followed a study Abu Bakar et al*.*^14^. The feedstock is converted using a biological process through hydrothermal pre-treatment and enzymatic hydrolysis to obtain C6 sugars. The details of conditions used in the process are shown in Supplementary Table S1. The EPFG was ground using a hammermill to increase the surface area for pre-treatment and enzymatic hydrolysis process. The ground biomass underwent hydrothermal pre-treatment at 120 °C for 60 min. The pre-treated EPFG was then subsequently subjected to enzyme hydrolysis process. A mixture of glucoamylase and cellulase enzyme was added to 10% solid-loading biomass. Glucoamylase was mainly used since this enzyme is required for starch hydrolysis. Description of the Eco invent database used as background process for glucoamylase were shown in Supplementary Table S2. The saccharification process was maintained at 60 °C for 72 h to yield 76% hydrolysis. Next, the solid and liquid fractions in the reactor were separated. The liquid fractions contained the desired product, which is glucose. However, the purification process was not considered since fermentation process does not require concentrated sugars^9^. In this scenario, a zero burdens approach from the cultivation phase was assigned to EPFG to focus on the impacts of glucose production through the bioconversion route.

#### Scenario 2 (S2)—waste utilisation as lignin residue

The solid residue obtained after saccharification was rich in lignin composition and could be revalorized^15^. In biorefinery, recycling waste generated from the process is essential to ensure maximum utilisation of biomass fractionation. Lignin residue demonstrated great potential for electricity generation^16^. Therefore, the solid residue with ~ 50 wt% moisture is combusted in a boiler for electricity generation to supply the glucose production plant^17^. After hydrolysis, the calorific value of the solid residue was 14.3 GJ/ton (data obtained from Biomass Technology Centre, UPM). The residue was incinerated in a combustor, boiler, and turbogenerator to produce electricity for the biorefinery plant. The system was assumed to produce 8–10 ton-steam/h at 300 °C, which is further converted to electricity^18,19^. In this study, the steam generated from the boiler was used only in an electricity generator, and this steam was not used for any other purpose in the process. The boiler efficiency is at 80%, and the turbogenerator efficiency of the turbine shaft conversion to electricity was assumed to be 85% following Humbird et al*.*^20^. Combustion of 1.6 tons of lignin residue generates 22.88 GJ of higher heating values. 1 GJ of heating value is equivalent to 277.8 kWh of electricity based on the theoretical energy equivalence^16^. Based on the efficiency boiler, 1 GJ of high heating values generates 188.9 kWh. Therefore, 4322.12 kWh of electricity is generated by combustion of the correspondingly generated lignin residue from producing one metric ton of glucose. In addition, the emissions produced from the boiler operation can be referred to Rathnayake et al*.*^21^ and shown in Supplementary Table S3. The electricity produced by lignin combustion is assumed to be used at the plant. The remaining excess electricity was sold back to the grid, providing a co-product credit.

#### Scenario 3 (S3)—considering agriculture and milling phase

The current practice of EPFG management in Malaysia is by disposal to landfill. Hence, scenario 3 is necessary if an economic value is given to EPFG in the future. The life cycle stages in this scenario include paddy cultivation, harvesting, paddy milling, and EPFG conversion process. Paddy cultivation was included in this study since the production of EPFG utilised the same input as quality grains, except the grain is deformed and/or immature, making it a type of waste removed during the milling process. The cultivation and milling inventory were gathered from the primary sources at the actual sites in Malaysia. The sites chosen for cultivation and milling inventory data were at Sungai Burong, Selangor, as one of the granary areas. The agriculture activities include land preparation, cultivation, maintenance, and harvesting, while the milling input includes drying and separation of certified seeds and EPFG. Input data included in this phase consist of seeds for cultivation, diesel for machineries, and agrochemicals for cultivation. The inventory data was collected through questionnaires, interviews, and expert judgement from the paddy industry players^14^. Since the EPFG is a waste available during paddy cultivation and was removed in paddy milling factory to separate quality and non-quality grains, an allocation factor of 0.24 was used for inventory data calculation^14^.

### Inventory and key assumptions

The inventory data for the three scenarios were calculated and analysed from primary and secondary sources. The results from the bioconversion process of EPFG to glucose were collected as primary data from laboratory-scale experiments and in previous research work from Biorefinery Complex, Universiti Putra Malaysia (UPM). The secondary information for input, such as enzyme production was obtained from the literature. In addition, the Ecoinvent database version 3.0 was used for other aspects, such as the production of chemicals and electricity. Furthermore, the data sources for agriculture and milling process were collected based on questionnaires, interviews with farmers and millers, literature, and Ecoinvent 3.0 databases. The emissions from co-generation were calculated based on^21^. The emissions and raw material consumption data were converted to a scale of 1 ton of glucose production. The details of the data inventory for all scenarios can be found in Table 1. The LCA studies were conducted based on the following assumption:

1. The paddy production was assumed to follow the average production per ha in Integrated Agriculture Development Area (IADA) paddy area, Malaysia^14^.
2. Herbicides and pesticides in agriculture phase are negligible and were considered as cut off criteria^10^.
3. Transportation of paddy to milling factory was assumed to be within the agriculture phase burden with 20 km distance.
4. Glucose production was assumed to be integrated within the milling factory; hence, no transportation of the biomass to the biorefinery was considered.
5. Purification phase for concentrated sugars was not included in the study; therefore, wastewater treatment was not considered in the bioconversion phase.
6. The CO_2_ emissions from biomass combustion processes were not considered according to the carbon–neutral rule.

**Table 1:** Inventory data of glucose production based on different scenarios.

	Scenario 1	Scenario 2	Scenario 3	Unit
Input—agriculture phase				
Organic amendment	–	–	0.03	ton
Limestone	–	–	0.01	ton
Diesel-leveller	–	–	11.65	L
Area	–	–	0.34	ha
Paddy seed	–	–	0.05	ton
Water	–	–	256.90	m3
Diesel-transplanting	–	–	4.11	L
Fertilizer	–	–	128.50	ton
Gasoline	–	–	7.54	L
Diesel for harvesting	–	–	8.91	L
Diesel for transportation	–	–	3.77	L
Input—milling phase				
Electricity	–	–	106.57	kWh
Diesel—drying	–	–	0.027	ton
Input—bioconversion system				
EPFG processing				
EPFG	2.60	2.60	2.60	ton
Electricity	78	78	78	kWh
Pre-treatment				
Electricity	261.63	261.63	261.63	kWh
Water	23.14	23.14	23.14	ton
Steam	0.50	0.50	0.50	ton
Enzymatic hydrolysis				
Electricity	62.41	62.41	62.41	kWh
Water	2.57	2.57	2.57	ton
Sodium acetate	0.07	0.07	0.07	ton
Acetic acid	0.03	0.03	0.03	ton
Enzyme-glucoamylase	52.90	52.90	52.90	L
Enzyme-cellulase	0.068	0.068	0.068	ton
Separation				
Electricity	25.20	25.20	25.20	kWh
Output				
Glucose	1.00	1.00	1.00	ton
Solid residue	1.60	1.60	1.60	ton
Electricity generation	–	4322.12	4322.12	kWh

### Life cycle impact assessment

The LCA results were obtained using ReCiPe methodology via the SimaPro v8.0 LCA software. ReCiPe methodology was used in this study because it had diverse impact categories that will assist policymakers in ensuring all possible consequences related to the glucose biorefinery scenarios^22^. The impact categories accounted for using the aforementioned method were climate change (CC), terrestrial acidification (TA), freshwater eutrophication (FE), human toxicity (HT), photochemical oxidant formation (POF), particulate matter formation (PMF), terrestrial ecotoxicity (TET), freshwater ecotoxicity (FET), fossil depletion (FD), and agricultural land occupation (ALO).

## Results and discussion

### Contribution analysis for each scenario

**Figure 2:**
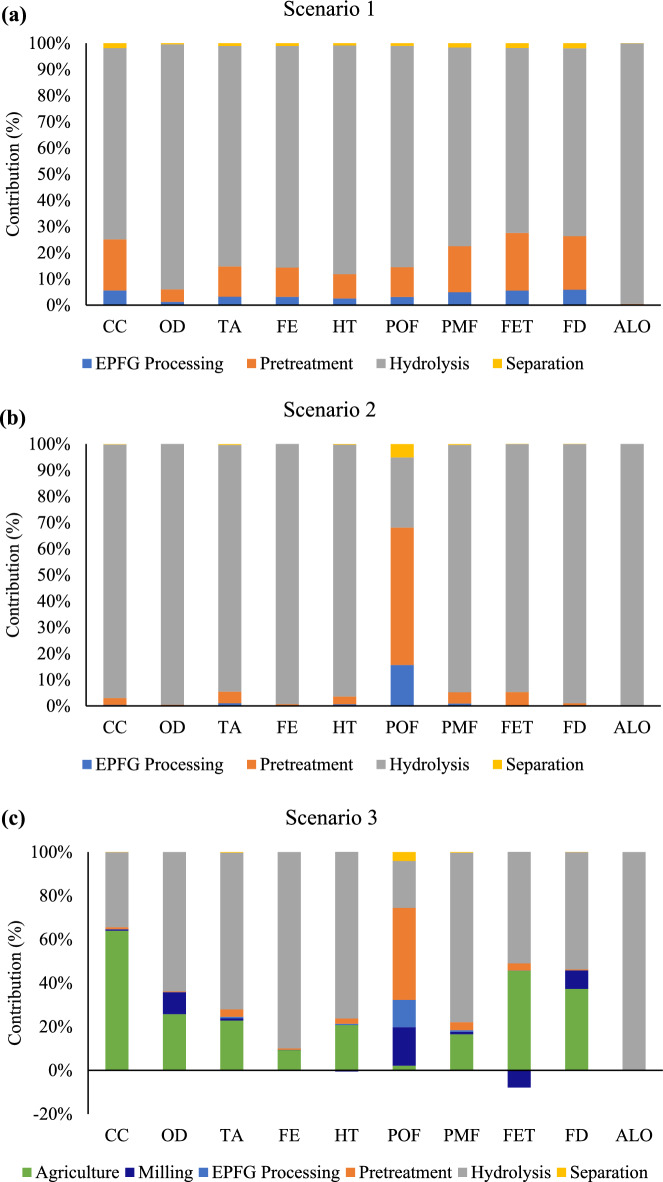
Contribution analysis for (**a**) scenario 1, (**b**) scenario 2, (**c**) scenario 3.

Figure 2 presents the 10 categories covered by the ReCiPe methodology. The environmental impact was presented as a contribution analysis and was divided according to the processes involved. A summary of each scenario’s environmental burdens is shown in Supplementary Table S4. From the data, it can be seen that hydrolysis is the main contributor in all categories for scenario 1 followed by pre-treatment process (Fig. 2a). The impact of the hydrolysis process is caused by the use of enzymes and chemicals for media preparation needed in the production of glucose. The chemical utilisation in hydrolysis process is for the production of buffer solution needed to stabilise the pH for enzyme hydrolysis. The contributions of enzyme input are significant in the hydrolysis process representing 70% or all impact categories. Therefore, the enzyme hydrolysis process of EPFG requires further attention as it is the hotspot in the glucose production system. Further research revealed that the production of enzymes used in EPFG hydrolysis was reported to cause significant environmental burdens^23,24^. The background activities of enzyme production have been analysed in this study, and it has been found that electricity consumption and agriculture activities are the main hotspots for this input. Furthermore, enzymatic hydrolysis was also shown to have the worst impact on ALO at 100%. The impacts of ALO are predominantly derived from the cultivation phase for enzyme production. The enzyme production required sugars and protein from potato juice and wheat/corn starch^23^. The use of these ingredients obtained from plant sources causes a high impact on ALO. This was also observed in an LCA study conducted by Subramanian et al*.*^25^ where the author reported that enzymes for glucose recovery from cotton polyester have the highest impact on land use at 49%. In addition, the pre-treatment phase contributes to more than 20% for CC and FET due to electricity consumption for operating hydrothermal pre-treatment. EPFG processing and separation phase account for less than 8% of the impact, with electricity consumption as the input.

Next, the environmental impact shows improvement when lignin residue is utilised in scenario 2 (Fig. 2b). In all impact categories, the ability to generate a high-quality energy carrier, such as electricity, from solid and liquid fractions was associated with significant environmental savings. For example, the hotspots for environmental burden have decreased for most of the impact categories, as shown in Supplementary Table S4. The pre-treatment phase shows contribution reductions due to the replacement of input from the current electricity mix using natural gas/coal to renewable energy^26^. Since hydrolysis and pre-treatment phases consume high electricity compared to other phases, significant impact reduction is observed in these phases, especially the hydrolysis phase.

Furthermore, compared with other categories, the POF category shows a different trend in scenario 2, with the pre-treatment phase having the highest impact contribution at 42.1%. The input related to POF is mainly due to energy input for operational processes. The POF category in this model depends on the formation of sulphur dioxide (SO_2_), carbon monoxide (CO), nitrogen dioxide (N_2_O), ammonium, and non-methane volatile organic compounds (NMVOC)^27^. This must also consider that hydrothermal pre-treatment of the biomass requires higher electricity for operation. Hence, even by changing grid electricity to renewable electricity, the input of biomass combustion is still responsible for POF impact^28^.

Agriculture management is crucial in the generation of EPFG. The generation of EPFG is dependent on environmental factors (climate), soil conditions, crop production practices, and postharvest production management^29^. For example, extreme weather conditions during the planting season cause production of low paddy quality, resulting in damaged paddy production. Scenario 3 was conducted to observe the impact of the agriculture and milling phases on the glucose production system. Adding the agriculture and milling phase within the boundary shows an increase in the contribution for all impact categories from scenario 2. The worst case is seen in CC, OD, FET, and FD impacts with more than 25% contribution. Agriculture activities encompass emissions to air, water, and soil due to the application of fertiliser and diesel. However, the increase in CC impact is mainly contributed by continuous flooding as paddy cultivation practice and fertiliser usage^14^. The total climate change impact is increased to 2236.04 kg CO_2_ eq in scenario 3 with a 64% contribution. The milling operations for drying and separating quality paddy with EPFG commonly involve diesel and electricity inputs; thus, this input contributes towards POF, OD, and FD. Since in scenario 3 the electricity has been replaced by renewable electricity through lignin combustion, the input responsible for these impact categories in the milling process is due to diesel usage for the drying process.

### Comparative assessment

**Figure 3:**
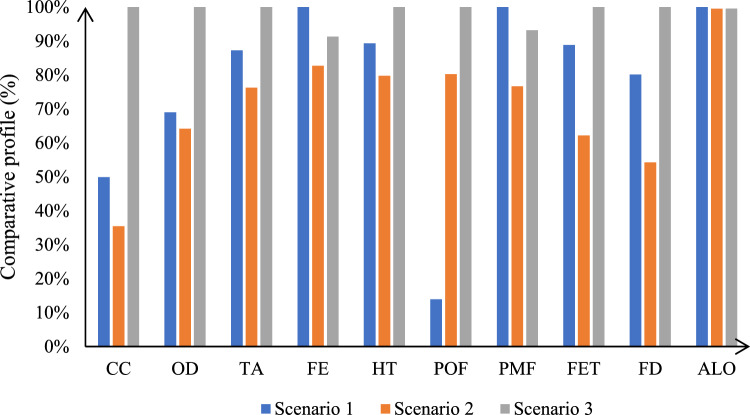
Comparative environmental profile of glucose production from EPFG.

Comparative profile among scenarios is shown in Fig. 3. The CC impact is less in S1 and with improvement in S2. The CC impact in S2 has been improved from 1130.37 to 803.49 kg CO_2_ eq with a 15% reduction from S1. The highest contribution for CC impact was shown in S3 due to additional input in agriculture phase. One of the main input contributors to emissions from S3 was due to fertilizer usage. Conventional rice in Malaysia relies heavily on synthetic fertilizer for nutrient inputs. The input fertilizer was assigned to quality grain and EPFG using mass allocations. Fertilization management is one factor that plays a major role in determining the amount of EPFG that can be removed in a sustainable system^29^. The use of fertilisers impacts CC categories as fertiliser is an important source of nutrient-related emissions (N_2_O, NO_x_, NH_3_, PO_4_)^30^. The agriculture phase contributes 1448.25 kg CO_2_ eq for CC impact to produce 1-ton glucose.

Lower impact contributions from S2 were observed for most of the impact categories. Replacement of fossil/coal combustion for energy with biomass combustion offers a cleaner production^31^. The glucose production system required 427 kWh of electricity; thus, 3895 kWh is produced as excess electricity used in the refinery. The remaining surplus electricity can be sold to the grid to replace coal-based electricity^32^. In an LCA study of orange peel waste in a biorefinery, Teigiserova et al*.*^33^ observed a decrease in CC impact when changing from a conventional energy mix in the biorefinery to renewable energy. Furthermore, utilisation of sugarcane bagasse as an energy source for feedstock processing in biofuel production provides environmental advantages since energy is produced more than energy required for the systems^34^.

Nevertheless, POF for S1 is much lower than the other scenarios. The increase in POF for S2 and S3 was due to the increased levels of NOx and SO2 emissions. The results also show that the nitrates and phosphorus emission levels in these scenarios increased due to the usage of biomass combustion for energy. According to Rathnayake et al*.*^21^, the emissions were related to the background input of biomass cultivation due to the usage of chemical fertilizers and herbicides/pesticides application, as well as heavy metals leaching into the environment. Despite that, for other impact categories, using EPFG as raw materials in the biorefinery platform is an effective option for reducing environmental concerns related to fossil resources and enhancing cleaner production chains.

### Comparison of damage assessment among different scenarios

Damage assessment (DA) was performed using the endpoint level to observe the environmental impacts of glucose production on human health (HH), ecosystem quality (ED), and resource availability (RA). The endpoint results are shown in Table 2. The base case impacted the HH, ED, and RA at 2.27E−03 DALY, 1.68E−05 species.yr, and 56.62 Dollar. Therefore, from the table, a reduced impact for all DA categories was observed when utilizing all EPFG fractionation as in scenario 2. The results show that using lignin residue to replace electricity grid mix reduced the DA. The DA of HH is contributed by OD, PMF, HT, and CC impact categories. An increase in chemicals, fertilizers, and electricity usage in scenario 3 causes additional environmental loads to these impacts. Other input was added when the model system was expanded to agriculture phase, which also caused the increase in resources. For this reason, less chemicals, more energy efficiency usage, and consideration of the use of renewable energy resources would result in a reduction in the impact categories value, thus, lowering the overall value of the damage associated with the activities^35^. Hence, it is observed that the results for DA having the lowest damage are at scenario 2 < scenario1 < scenario 3.

**Table 2:** Damage assessment at the endpoint level for glucose production.

Damage category	Unit	Scenario 1	Scenario 2	Scenario 3
Human health	DALY	2.27E−03	1.67E−03	3.83E−03
Ecosystems	species.yr	1.68E−05	1.41E−05	2.57E−05
Resources	$	56.62	39.3	69.54

### Allocation results

Allocation results were applied to S1 as the base case to assess the effects on the environmental outcomes using two allocation methods. The allocation was distributed among products in S1, glucose, and lignin residue. Mass allocation and economic allocation at a ratio of 1:1.7 and 2.2:1 was applied to glucose and lignin residue, respectively (see Supplementary Table S5). The results obtained are depicted in Table 3. The outcomes per functional unit of economic allocation are about 54% higher than those of mass allocation for glucose production in CC impact. This is due to the price of glucose being much higher since the conversion process uses enzymes, which is deemed expensive in the hydrolysis phase^36^. Hence, this explains the economic allocation results were much higher than the mass allocation for glucose production. Most studies acknowledge mass allocation rather than the economic allocation method since the price of a product keeps changing depending on various factors^36^. The raw materials cost is the primary component contributing to higher glucose prices^37^. Nevertheless, the significant impact of glucose valorisation from EPFG using the mass-based allocation method was the CC, HT FD, and ALO. This is mainly due to the type of electricity mix from fossil/coal-based input.

**Table 3:** Environmental of glucose production based on allocation method.

Impact category	Unit	Economic allocation	Mass allocation
Climate change	kg CO_2_ eq	962.44	794.27
Ozone depletion	kg CFC-11 eq	4.25E−05	3.79E−05
Terrestrial acidification	kg SO_2_ eq	5.86	5.15
Freshwater eutrophication	kg P eq	0.08	0.07
Human toxicity	kg 1,4-DB eq	77.30	67.18
Photochemical oxidant formation	kg NMVOC	3.09	2.68
Particulate matter formation	kg PM10 eq	2.09	1.76
Freshwater ecotoxicity	kg 1,4-DB eq	1.09	0.88
Fossil depletion	kg oil eq	266.87	214.07
Agricultural land occupation	m2a	373.88	371.66

### Sensitivity and uncertainty analysis

**Figure 4:**
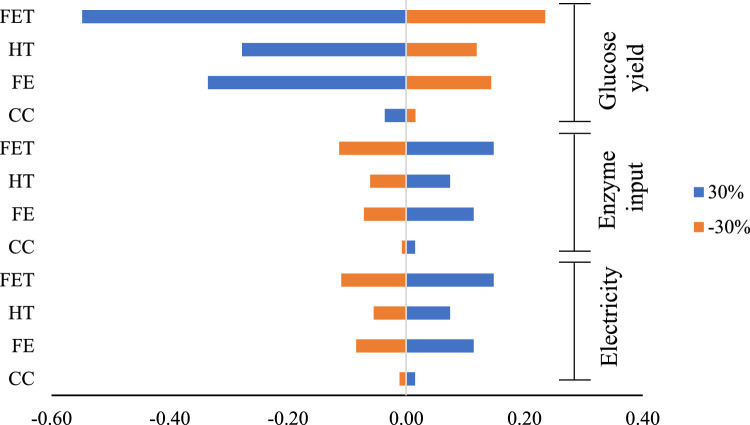
Sensitivity analysis for glucose production based on scenario 1.

One-way sensitivity analysis was performed on scenario 1 as the base case for the study. Normalised data were used to differentiate between base case and sensitivity results at ± 30% input variables. The data selection allows comparing all impact categories using the same scale within this scenario^38^. Among all input variables, electricity, enzyme input, and glucose yield have been shown to significantly affect the environmental load. Using a tornado diagram, the sensitivity results are shown in Fig. 4. Figure 4 displays the most influential parameter changes on FET, HT, FE and CC impacts. Other impact changes compared to the base case scenario are presented in Supplementary Table S6. From Fig. 4, the reduction in the enzyme and electricity input caused a decrease in the impact category, especially on FET, FE, and HT.

In contrast, the CC impact changes are relatively small. Since the input affecting this impact was mainly due to glucoamylase production and agriculture phase as shown in scenario 3, hence, changes in the present input variables are less significant for CC impact. However, an increase in the input variables is expected to increase the environmental load of glucose production. Interestingly, the sensitivity analysis on glucose yield seems to have better performance towards the environment when the product yield is increased. This is because an increase in glucose yield means increased hydrolysis efficiency in the bioconversion process. The significant positive effect of product yield on environmental factors was also discussed by Larnaudie et al*.*^39^. The author showed that an increase in ethanol yield correlated with a decrease in environmental impacts since the input conversions value was maintained. Therefore, it can be said that with all process variables at constant, the increased product yield leads to a decreased environmental impact as better process efficiency is achieved. A reported study of glucose production from cotton shows that the CC impact is more sensitive when changes of input occur during the pre-treatment process^25^. The author reported that energy and urea are the main input that causes an impact on CC.

Uncertainty was performed along with the sensitivity analysis using Monte Carlo analysis to validate the robustness of the environmental impact categories. The Monte Carlo analysis was carried out in Excel using 1200 replications based on the uniform distribution for the random number generation of the variables within the uncertainty range^40^. The uncertainty results are depicted in Table 4. The environmental impacts varied within 10–11% of the base value when enzymes and glucose yield input varied at ± 30%. The variation of impact categories is much lower at 5–8% when electricity input is changed. A low coefficient variation and standard deviation suggest that the results are reliable within the range of the values considered. Therefore, the results of the base case can be a good representative of the model.

**Table 4:** Uncertainty analysis of environmental impacts of glucose production from EPFG in S1 based on input changes.

Input	Impact categories	Base case	Mean	Median	Standard deviation	Coefficient variation (%)	Confidence level (95.0%)
Electricity	FET	2.375	2.386	2.386	0.133	5	0.007
	HT	1.204	1.209	1.209	0.064	5	0.004
	FE	1.381	1.464	1.465	0.100	7	0.006
	CC	0.157	0.158	0.158	0.013	8	0.001
Enzyme	FET	2.375	2.452	2.447	0.264	10	0.014
	HT	1.204	1.249	1.251	0.135	11	0.008
	FE	1.486	1.508	1.500	0.162	11	0.009
	CC	0.157	0.163	0.163	0.016	10	0.001
Glucose yield	FET	2.375	2.471	2.473	0.257	10	0.015
	HT	1.204	1.255	1.251	0.138	11	0.008
	FE	1.230	1.509	1.509	0.156	10	0.009
	CC	0.157	0.163	0.163	0.017	10	0.001

### Comparison among other glucose production study

Comparing all categories is quite impossible since most of the published studies lack similarities between the LCA methodology. Furthermore, most of the literature focuses on the final product, such as bioethanol or bio-based product, compared to intermediate products such as fermentable sugars^6,9,41,42^. However, Bello et al*.*^6^ have discussed the possibilities of environmental impacts from first- and second-generation sugars in the European region that can be used as a reference and guidance for Malaysian glucose production. In the paper, eucalyptus converted to glucose through dilute acid pre-treatment has 2.5 kg CO_2_ eq/kg glucose for CC impact^6^. In another study, Baral et al*.*^43^ modelled the glucose production from sugarcane bagasse through alkaline pre-treatment, resulting in an impact of 1.57 kg CO_2_ eq/kg glucose. The results obtained from both studies are higher than this study, with an impact of 1.08 kg CO_2_eq/kg glucose as in scenario 1. The results in this study are much lower since no chemicals are required in the pre-treatment steps. Furthermore, when comparing with other impact categories, Moreno et al*.*^42^ showed that the production of 1 kg glucose from corn starch using enzymatic routes had an impact of 5.6E−7 kg CFC-11 eq for OD, 1.48 kg oil eq for FD, and 0.354 kg 1,4-DBeq for HT. These findings also have higher impact compared to this study. The results obtained in this study using EPFG as feedstock through enzymatic routes are at 4.55E−8 kg CFC-11 eq for OD, and 0.31 kg oil eq for FD. While for HT, the impact is slightly higher in this study at 0.392 kg 1,4-DBeq for HT, due to enzymatic hydrolysis phase.

### Recommendation of EPFG management to glucose production in Malaysia perspectives

The Malaysian government provides vital support and involvement in ensuring optimum paddy production and farmers’ well-being. Nevertheless, various factors, especially climate conditions, still lead to postharvest losses through EPFG generation. The utilisation and exploitation of agricultural waste have been widely discussed under the circular economy (CE) concept^44^. Therefore, the use of EPFG for glucose production provides an effective option for paddy milling waste management. Through the biorefinery concept, not only can glucose be produced for various purposes, such as animal additives and fermentation intermediates, but it can also generate electricity by replacing fossil-derived energy, which can also be utilised by the paddy mill. Based on the sensitivity results, an increase in glucose yield reduces the environmental impact. Hence, improvements in the conversion process should be studied using different techniques to increase glucose yield and reduce enzyme usage. Not only that, but the biorefinery systems have also been shown to help mitigate climate change, reduce reliance on imported fossil fuels, and improve cleaner manufacturing chains using local and renewable resources^45^. Sustainable growth for the biomass industry requires a balance between social, environmental, and economic factors. To enhance the project development of EPFG to glucose, techno-economic analysis is crucial to ensure profitability of a project. As shown in S2, using solid residue for generating bioenergy is recognised as a promising process for effective waste management by making use of most of the biomass, as in the biorefinery concept. However, the question is whether the co-production of glucose from EPFG does generate revenue for the plant and still makes the conversion of waste to product scheme economically viable. Hence the economic assessment is recommended in future studies. Another aspect worth looking into is the social impact of converting waste into a product in a biorefinery platform. Malaysians must recognise the measures to advance the biomass industry to address global energy security and climate change challenges, as well as foster synergy with the agricultural sectors to increase national income^46^. Additionally, all stakeholders should be involved in supporting sustainable development and CE in Malaysia. For example, the government, agriculture agencies, and industrial players need to work together to ensure sustainability in the paddy industry by providing competent and easily accessible advisory services, supporting research and development, and providing more capital support. Conclusively, paddy milling waste utilisation should also be introduced to all relevant stakeholders as a co-benefit of policies, practices, and actions concerning circular economy activities in Malaysia.

## Conclusions, future work, and limitations

A cradle-to-gate LCA was performed for glucose production from EPFG within the Malaysian context as an alternative for managing paddy processing waste. Hence, an environmental assessment was analysed in three scenarios based on system expansion. It was found that the environmental hotspot for glucose production was contributed by enzymatic hydrolysis phase, with enzyme production as the main input impacting the environment. Improved environment loads were seen in scenario 2 when all EPFG fractionation was utilized. The electricity generation was used back by the glucose plant leading to saving emissions by replacing coal-based electricity. The cultivation phase contributes considerably to CC, OD, and FD impacts due to flooded rice practices and fertilizer input consumption. In addition, a sensitivity analysis was performed on scenario 1, whereby it can be seen that an increase in glucose yield leads to a significant reduction of environmental impacts. Therefore, future research should focus on improving the bioconversion process to yield high concentrations of glucose from the system. Moreover, since enzyme production is among the hotspot of the study, future research should be conducted for utilising enzymes produced from other sources, such as mixed food waste^47^. Furthermore, the benefits on emission potential as determined in this study can be used as the basis for policy promotion, e.g., granting subsidies for biorefinery integrated within paddy milling factories when utilising EPFG and selling it to replace other market products such as fuel and feed. Therefore, generating monetized value through techno-economic analysis is necessary to strengthen the benefits of waste to value-added products, especially in the paddy industry.

In the case of limitations of the study, the data sources obtained for the conversion process were based on laboratory work which differs from when an actual glucose plant is applied in the paddy industry. The performance is expected to vary when a process is scaled up from the laboratory to the pilot and commercial scale. Hence, a realistic figure should be obtained as comprehensively as possible to yield environmental results for the Malaysian scenario. Consideration of uncertainty sources is critical in the LCA, which may impact the outcome of the study. For example, input on electricity production data and assumption of co-product allocation can result in less inventory accuracy and lead to uncertainties. Other aspects that should be considered as LCA limitations within biorefinery scope are the transparency of an LCA study, data gaps such as enzyme database, inconsistency in methodological choices such as system boundary, and functional unit. These limitations are challenging for LCA practitioners to obtain accurate data and compare it to LCA results from different studies. Nevertheless, the results of this study should provide a basis for the improvement of waste management, especially in the Malaysia paddy industry. In addition, the study aligns with Twelfth Malaysia Plan 2021-2025 to support waste documentation to -to-wealth potential and could provide national database access, especially for LCA practices.

### Supplementary Information

Supplementary Information.

## Data Availability

The data that supports the findings of this study are available in the supplementary material of this article.
