# Supplementary material for: Environmental impact assessment of rice mill waste valorisation to glucose through biorefinery platform
Source: Sci Rep. 2023 Sep 7;13:14767. doi: 10.1038/s41598-023-28487-2 (PMC10484976; doi:10.1038/s41598-023-28487-2)
Supplement: Supplementary file 1 — Supplementary Information. [file 41598_2023_28487_MOESM1_ESM.pdf]

SUPPLEMENTARY MATERIAL FOR

Environmental impact assessment of rice mill waste valorisation to glucose through biorefinery platform

Nurul Ain Abu Bakar[1, 2], Ahmad Muhaimin Roslan[1, 5*], Mohd Ali Hassan[1], Mohammad Hariz Abdul Rahman[2], Khairul Nadiah Ibrahim[3], Muhammad Daaniyall Abdul Rahman[4] and Rozyanti Mohamad[3]

[1]Department of Bioprocess Technology, Faculty of Biotechnology and Biomolecular Sciences, Universiti Putra Malaysia, 43400 UPM Serdang, Selangor

[2]Agrobiodiversity and Environment Research Centre, Malaysia Agriculture Research and Development Institute, Persiaran MARDI-UPM, 43400 Serdang, Selangor

[3]Universiti Kuala Lumpur, Malaysian Institute of Chemical and Bioengineering Technology, Lot 1988 Bandar Vendor, Taboh Naning, 78000 Alor Gajah, Melaka

[4]School of Business and Economics, Universiti Putra Malaysia, 43400 UPM Serdang, Selangor

[5]Biopolymers and Derivatives Laboratory, Institute of Tropical Forestry and Forest Product, Universiti Putra Malaysia, 43400 Serdang, Selangor

*Corresponding author: ar_muhaimin@upm.edu.my

Department of Bioprocess Technology, Faculty of Biotechnology and Biomolecular Sciences, Universiti Putra Malaysia, 43400 UPM Serdang, Selangor, Malaysia

Tel: +603-97698054

Table S1 Composition and process conditions of glucose production [1].

Feedstock	Composition (wt%)	Process operations
Empty and partially filled paddy grain	Starch: 14%<br>Cellulose: 32.6%<br>Hemicellulose: 14.3%<br>Lignin: 12.3%<br>Moisture content: 14%	Hydrothermal pretreatment: 120ºC, 60 minutes<br>Enzymatic hydrolysis: 60 U/g glucoamylase, 10 FPU/g cellulase, 60ºC, 150 rpm.

Table S2 Ecoinvent data for the production of 1 kg glucoamylase [2].

Input	Amount	Unit
Potato starch, at plant [DE]	4.17	kg
Heat, unspecified, in chemical plant [RER]	8.3	MJ
Electricity, medium voltage, production MY, at grid [MY]	6.3	kWh

Table S3 Emission factors for lignin residue combustion [3]

Emissions	Residue (mg/MJ)
CO_2	-
CO	90.0
CH_4	3.1
NO_x	81.0
N_2O	0.8
SO_2	1.9
NMVOC	5.1
Particulates	61.0

Table S4 Contribution analysis - Characterization of the LCIA midpoint of glucose production for three different scenarios.

Impact categories	Unit	Scenario 1	Scenario 2	Scenario 3
CC	kg CO_2 eq	1083.40	757.72	2236.04
OD	kg CFC-11 eq	4.55E-05	4.22E-05	1.83E-05
TA	kg SO_2 eq	6.19	5.39	7.25
FE	kg P eq	0.42	0.31	0.32
HT	kg 1,4-DB eq	392.09	314.18	377.02
POF	kg NMVOC	3.43	20.19	25.11
PMF	kg PM_{10} eq	2.36	1.80	2.25
FET	kg 1,4-DB eq	10.24	8.11	9.66
FD	kg oil eq	307.52	204.32	391.83
ALO	m_2a	300.42	298.61	341.01

Table S5 Mass and economic allocation of co-product.

Output	Mass (t/t)	%	Economic value (€/t)	%
Glucose	1	37	330[a]	69
Lignin residue	1.7	63	150[b]	31

[a]Brant et al., [4]

[b]Obydenkova, et al. [5]

Table S6 Sensitivity analysis for glucose production (normalisation data).

Impact categories	Base case	Electricity 30.0%	Electricity -30.0%	Glucoamylase input 30%	Glucoamylase input -30%	Glucose yield 30%	Glucose yield -30.0%
CC	0.157	0.172	0.146	0.181	0.150	0.121	0.173
OD	0.001	0.001	0.001	0.001	0.001	0.001	0.001
TA	0.162	0.171	0.155	0.193	0.153	0.125	0.178
FE	1.454	1.568	1.369	1.692	1.382	1.118	1.597
HT	1.204	1.278	1.149	1.406	1.143	0.926	1.323
POF	0.060	0.064	0.058	0.072	0.057	0.046	0.066
PMF	0.168	0.182	0.157	0.195	0.159	0.129	0.184
FET	2.375	2.523	2.266	2.753	2.262	1.827	2.610
FD	0.239	0.263	0.221	0.271	0.229	0.184	0.262

Table S7. List and description of main dataset that were included in life cycle inventory

Phase	Dataset	Value	Unit	Remarks
	**Products**			
	Glucose Scenario	1	ton	
	[a]**Avoided products**			
	Electricity, medium voltage {MY}| market for | Alloc Def, S	4	kWh	
	**Materials/fuels**			
Agriculture and milling phase	[b]National data			Refer to Abu Bakar et al., (2022)

Bioconversion process	Tap water, at user {RoW}| market for | Conseq, U	23142.9	kg	
	Glucoamylase	52.9	kg	Refer to Table S2
	Tap water, at user {RoW}| market for | Conseq, U	2571.4	kg	
	Cellulase	0.1	kg	
	Acetic acid, without water, in 98% solution state {GLO}| market for | Alloc Def, S	25.7	kg	
	Sodium {GLO}| market for | Alloc Def, S	70.3	kg	
	**Electricity/heat**			
	Electricity, medium voltage {MY}| market for | Alloc Def, S	101.4	kWh	Processing
	Electricity, medium voltage {MY}| market for | Alloc Def, S	261.63	kWh	Pretreatment
	Electricity, medium voltage {MY}| market for | Alloc Def, S	81.13	kWh	Enzymatic
	Electricity, medium voltage {MY}| market for | Alloc Def, S	32.76	kWh	Separation
	**[a]Waste to treatment**			
	Lignin residue	1.6	ton	Refer to Rathnayake et al., (2018)

a Inventory included in S2 and S3
b Inventory included in S3

References

1. Abu Bakar, N.A., Roslan, A.M., Hassan, M.A., Abdul Rahman, M.H., Ibrahim, K.N., Abdul Rahman, M.D., & Mohamad, R. Development of life cycle inventory and greenhouse gas emissions from damaged paddy grain as fermentation feedstock: A case study in Malaysia. *Journal of Cleaner Production*, 354, 131722. DOI: https://doi.org/10.1016/j.jclepro.2022.131722 (2022).

2. Ahmadi, A., Severac, E., Monties, N., Claverie, M., Remaud-Simeon, M., Moulis, C., & Tiruta-Barna, L. An eco-design approach for an innovative production process of low molar mass dextran. *Green chemistry* **21**, 4512-4531 (2019).

3. Rathnayake, M., Chaireongsirikul, T., Svangariyaskul, A., Lawtrakul, L. & Toochinda, P. Process simulation based life cycle assessment for bioethanol production from cassava, cane molasses, and rice straw. *J. Clean. Prod.* **190**, 24–35 (2018).

4. Brandt, K.L., Gao, J., Wang, J., Wooley, R.J., & Wolcott, M. Techno-economic analysis of forest residue conversion to sugar using three-stage milling as pretreatment. *Front. Energy Res*. DOI: https://doi.org/10.3389/fenrg.2018.00077 (2018).

5. Obydenkova, S.V., Kouris, P.D., E.J.M., Smeulders, D.M.J., van der Meer, Y., & Boot, M.D. Industrial lignin from 2G biorefineries – Assessment of availability and pricing strategies. *Bioresource Technology*, 291,121805. DOI: https://doi.org/10.1016/j.biortech.2019.121805 (2019).
